# Light-Intensity-Dependent Control of Collagen Hydrogel Properties via Riboflavin Phosphate-Mediated Photocrosslinking

**DOI:** 10.3390/ma18040828

**Published:** 2025-02-14

**Authors:** Seungyeop Yoo, Won-Gun Koh, Hyun Jong Lee

**Affiliations:** 1 Department of Chemical, Biological and Battery Engineering, Gachon University, 1342 Seongnam-daero, Seongnam-si 13120, Republic of Korea; tldrjqnl@naver.com; 2 Department of Chemical and Biomolecular Engineering, Yonsei University, 50 Yonsei-ro, Seodaemun-gu, Seoul 03722, Republic of Korea

**Keywords:** collagen, photocrosslinking, light intensity, riboflavin phosphate, hydrogel

## Abstract

While photocrosslinked collagen hydrogels show promise in tissue engineering, conventional approaches for property control often require complex chemical modifications or concentration changes that alter their biochemical composition. Here, we present the first systematic investigation of light-intensity-dependent control in riboflavin phosphate (RFP)-mediated photocrosslinking as a novel, single-parameter approach to modulate hydrogel properties while preserving native biochemical environments. We systematically investigated the effects of varying light intensities (100 K, 50 K, and 10 K lux) during hydrogel fabrication through comprehensive structural, mechanical, and biological characterization. Scanning electron microscopy revealed unprecedented control over network architecture, where higher light intensities produced more uniform and compact networks, while swelling ratio analysis showed significant differences between 100 K lux (246 ± 2-fold) and 10 K lux (265 ± 4-fold) conditions. Most significantly, we discovered that intermediate intensity (50 K lux) uniquely optimized mechanical performance in physiological conditions, achieving storage modulus of about 220 Pa after 24 h swelling, compared to about 160 and 109 Pa for 100 K and 10 K lux conditions, respectively. Remarkably, cellular studies using NIH/3T3 fibroblasts demonstrated that lower light intensity (10 K lux) enhanced cell proliferation by 2.8-fold compared to 100 K lux conditions after 7 days of culture, with superior cell network formation in both 2D and 3D environments. This groundbreaking approach establishes light intensity as a powerful single parameter for precise control of both mechanical and biological properties, offering a transformative tool for tailoring collagen-based biomaterials in tissue engineering applications.

## 1. Introduction

Collagen, the most abundant protein in vertebrates, plays a crucial role in maintaining the structural and mechanical integrity of tissues [1]. Its unique properties, including inherent biocompatibility, biodegradability, and cell-binding domains, make it an attractive material for various biomedical applications such as tissue engineering, drug delivery, and regenerative medicine [2]. As the most abundant protein component of the natural extracellular matrix (ECM), collagen’s structural and biochemical properties make it an ideal biomaterial, particularly in the form of hydrogels that can mimic the three-dimensional tissue environment [3].

However, unmodified collagen hydrogels often exhibit limited mechanical properties and rapid degradation, which restrict their practical applications [4,5]. These limitations necessitate the development of effective strategies to enhance and control the physical properties of collagen-based materials [6,7,8,9]. Traditional approaches have relied on varying collagen concentration to modify mechanical properties [10]. However, this method simultaneously alters both the mechanical strength and the amount of cell-interactive components, making it difficult to isolate the specific effects of mechanical properties on cellular behavior [11,12,13]. Furthermore, concentration-based modification requires the preparation of multiple distinct solutions, increasing complexity and reducing experimental efficiency [14].

Among various approaches for modifying collagen properties, photocrosslinking using riboflavin-5′-phosphate (RFP) has emerged as a promising strategy [15,16,17]. While chemical crosslinking agents such as glutaraldehyde and carbodiimides have been widely used, they often present concerns regarding residual cytotoxicity and potential inflammatory responses [18]. Furthermore, compared to other photoinitiators like rose bengal and methylene blue, RFP demonstrates superior photocrosslinking efficiency and broader absorption spectrum in the visible range [19]. RFP, a phosphorylated form of vitamin B2, offers several advantages as a photoinitiator: enhanced water solubility, excellent biocompatibility, and FDA approval status [20,21]. Under blue light exposure (380–500 nm), RFP generates reactive oxygen species that facilitate the formation of covalent crosslinks between collagen molecules, resulting in stable hydrogel networks [19,22].

The photocrosslinking approach has been successfully applied to various natural polymer-based hydrogels beyond collagen. For instance, gelatin methacrylate (GelMA) hydrogels have demonstrated tunable mechanical and biological properties through light-mediated crosslinking [23,24]. Hyaluronic acid-based photocrosslinkable systems have shown promise in tissue engineering applications due to their controllable degradation rates [25,26]. These studies collectively demonstrate the versatility of photocrosslinking in natural polymer modification, while highlighting the unique advantages of collagen-based systems in terms of their innate biological properties.

Compared to chemical modification methods, light-mediated crosslinking presents distinct advantages: minimal use of potentially toxic chemicals, spatiotemporal control over the crosslinking process, and the ability to modify material properties without altering the chemical composition [27,28,29]. In particular, modulating light intensity during photocrosslinking offers a unique approach to control mechanical properties while maintaining consistent collagen concentration and biochemical composition. This method enables the fabrication of various hydrogels from a single precursor solution, significantly enhancing experimental efficiency and reproducibility [29,30]. Furthermore, it allows for the isolated study of mechanical effects on cellular behavior by eliminating variations in the concentration of cell-interactive components [31,32,33].

This study systematically investigates the influence of light intensity during fabrication on the properties of photocrosslinked collagen hydrogels. By examining hydrogels fabricated under three distinct light intensities (100 K, 50 K, and 10 K lux), we aim to establish correlations between fabrication parameters and functional outcomes. Through comprehensive structural analysis, mechanical characterization, and evaluation of cellular responses in both 2D and 3D environments, this research demonstrates a straightforward strategy for tailoring hydrogel characteristics through light intensity modulation. The findings provide valuable insights into optimizing collagen-based biomaterials for specific biomedical applications without requiring complex chemical modifications.

## 2. Materials and Methods

### 2.1. Materials

All chemicals and solvents were received from manufacturers without further purification unless otherwise noted. Mouse embryonic fibroblast (NIH/3T3) cells were obtained from the Korean Cell Line Bank (Seoul, Republic of Korea). Type I collagen solution (6 mg/mL, purity > 99%) was purchased from Advanced Biomatrix (Carlsbad, CA, USA). Dulbecco’s phosphate-buffered saline (DPBS), Dulbecco’s modified Eagle’s medium (DMEM), fetal bovine serum (FBS), 100× penicillin-streptomycin, and trypsin-ethylenediaminetetraacetic acid (trypsin-EDTA) solution were supplied by WelGene (Gyeongsan, Republic of Korea). Phosphate-buffered saline (PBS, pH 7.4), 3-(4,5-dimethylthiazol-2-yl)-2,5-diphenyltetrazolium bromide (MTT), 10X PBS, and LIVE/DEAD™ Viability/Cytotoxicity Kit were acquired from Thermo Fisher Scientific (Waltham, MA, USA). Riboflavin 5′-phosphate sodium salt hydrate (RFP, purity 73.0–79.0%) and sodium hydroxide solution were purchased from Sigma Aldrich (St. Louis, MO, USA). Dimethyl sulfoxide (DMSO) was obtained from Duksan (Seoul, Republic of Korea).

### 2.2. Hydrogel Matrix Preparation

Collagen hydrogels were fabricated using a photocrosslinking approach under three distinct light intensity conditions (100 K, 50 K, and 10 K lux). Light intensity was measured and calibrated using a digital light meter (UA-962, UYIGAO, Shenzhen, China) positioned at the sample surface level. The light-source-to-sample distance was fixed at 40 mm using a standardized positioning system. Light intensity variation across the illumination area was found to be less than 5%. The preparation protocol began with the dilution of stock collagen solution (6 mg/mL) to a working concentration of 3 mg/mL using a combination of 10× PBS, NaOH for pH adjustment (final pH 7.4), and distilled water at 4 °C. The photoinitiator RFP was incorporated as a 1% (*w*/*v*) solution in PBS to achieve a final concentration of 0.01% (*w*/*v*). The pre-gel solution (200 μL) was dispensed into cylindrical molds and exposed to blue light (wavelength: 450 nm) for 1 min at the specified light intensities to initiate crosslinking.

### 2.3. Characterization of Hydrogel

Microstructural analysis was performed using a scanning electron microscope (SEM, SU8600, Hitachi, Tokyo, Japan) at the Smart Materials Research Center for IoT, Gachon University, Republic of Korea. Sample preparation involved rapid freezing in liquid nitrogen followed by fracturing to expose internal cross-sections. The specimens were then lyophilized and sputter-coated with platinum under vacuum prior to imaging.

Hydrogel swelling properties were quantified by measuring mass changes after PBS immersion. Samples prepared in mini Petri dishes were submerged in 5 mL PBS and incubated at 37 °C for 24 h. Following careful removal of excess PBS, the swollen mass (*W*_w_) was recorded. The swelling ratio was determined relative to the dried mass (*W*_d_) according to the following equation:

(1) $$swelling\;ratio\;\left(fold\right)=\frac{W_{w}-W_{d}}{W_{d}}$$

The mechanical characteristics of the photocrosslinked hydrogels were analyzed using a rheometer (MCR92, Anton Paar, Graz, Austria) with frequency sweep measurements. Storage modulus (G′) and loss modulus (G″) were measured under physiological conditions (37 °C) using a 1% fixed strain, with frequency ranging from 0.1 to 10 Hz to evaluate viscoelastic behavior and network stability.

### 2.4. Cell Culture and Cytotoxicity Assay

For cellular studies, NIH/3T3 fibroblasts were maintained in high glucose DMEM supplemented with 10% FBS and 1% penicillin-streptomycin at 37 °C in a humidified atmosphere with 5% CO_2_. Cells were expanded in T-75 flasks until reaching 90% confluence. Cell viability was evaluated using two complementary approaches.

Metabolic activity was assessed via MTT assay in both 2D and 3D culture formats. For 2D studies, cells were seeded at 1 × 10^4^ cells per well in 24-well plates. For 3D culture, cells were encapsulated within hydrogels at a density of 1 × 10^4^ cells per 200 µL. At designated time points (days 1, 4, and 7), MTT solution was added followed by 1 h incubation. The resulting formazan crystals were solubilized in DMSO, and absorption was measured at 540 nm using a microplate reader (Epoch, BioTek, Winooski, VT, USA).

Live/dead fluorescence staining was employed to visualize cell viability. A staining solution containing calcein acetoxymethyl ester (Calcein AM) and ethidium homodimer-1 (EthD-1) (20 µL and 5 µL, respectively, in 15 mL DPBS) was prepared fresh. Culture samples were immersed in the staining solution with format-specific incubation times: 1 h for 2D cultures and 2 h for 3D cultures. After PBS washing, fluorescence imaging was performed using an EVOS M5000 imaging system (Thermo Fisher Scientific, Waltham, MA, USA).

### 2.5. Statistical Analysis

The results are expressed as means with standard deviations (SD). For swelling ratio measurements and MTT assays (both 2D and 3D), experiments were conducted with 3 independent samples per group. Statistical analyses were performed using GraphPad Prism version 10 (GraphPad Software, Inc., San Diego, CA, USA). For comparisons between two groups, Student’s *t*-test was used. For multiple group comparisons, one-way or two-way analysis of variance (ANOVA) was performed followed by Tukey’s post-hoc test. Differences were considered statistically significant when *p* < 0.05 (* *p* < 0.05, ** *p* < 0.01, *** *p* < 0.001, **** *p* < 0.0001).

## 3. Results and Discussion

### 3.1. Stable Formation of Photocrosslinked Collagen Hydrogels Under Different Light Intensities

**Figure 1:**
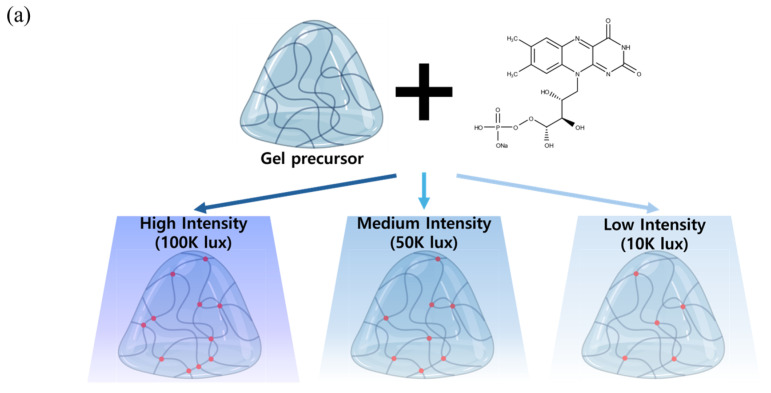

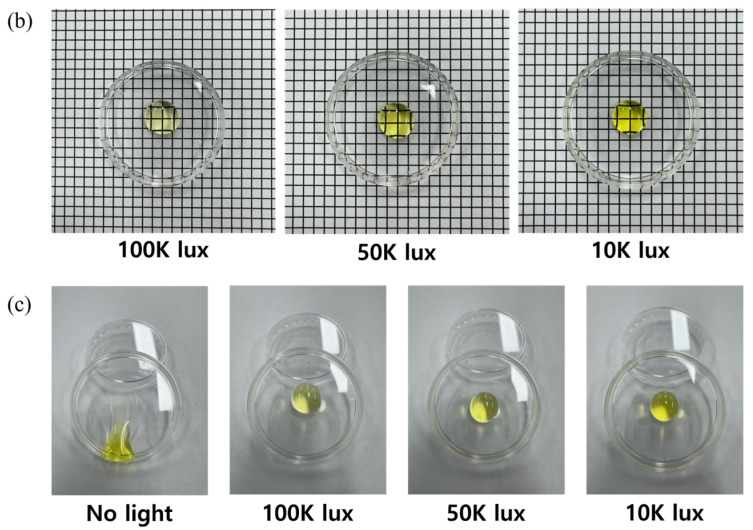
Fabrication process and characterization of photocrosslinked collagen hydrogels under different light intensities. (**a**) Schematic illustration of collagen hydrogel fabrication under varying blue light intensities. (**b**) Visual assessment of hydrogel formation showing transparency changes and RFP photobleaching over time. (**c**) Macroscopic images demonstrating hydrogel stability.

Collagen molecules undergo self-assembly to form triple helical structures under physiological conditions (37 °C, pH 7), naturally creating hydrogels through temperature and pH-mediated processes [34]. However, these self-assembled hydrogels typically exhibit limited mechanical stability and rapid degradation. To address these limitations, we implemented a photocrosslinking strategy using RFP as a photoinitiator under blue light irradiation (Figure 1a). This approach allows for controlled crosslinking under mild conditions while maintaining collagen’s inherent biocompatibility [35,36].

The photocrosslinking mechanism involves both Type I and Type II photochemical processes under blue light exposure (380–500 nm). In Type I reactions, excited RFP directly interacts with collagen molecules, while in Type II reactions, RFP produces reactive oxygen species (ROS), particularly singlet oxygen [19]. These ROS primarily target specific amino acid residues in collagen, including histidine, proline, lysine, and tyrosine, leading to the formation of covalent crosslinks. By modulating the blue light intensity (100 K, 50 K, and 10 K lux), we could systematically control the rate of ROS generation and, consequently, the crosslinking density of the hydrogels. At higher light intensities (100 K lux), the rapid generation of ROS leads to more uniform and dense crosslinking, while lower intensities (10 K lux) result in a more gradual and spatially varied crosslinking pattern. The progression of the crosslinking reaction was effectively monitored through the photobleaching of RFP, which gradually loses its characteristic yellow color upon blue light exposure due to photodegradation (Figure 1b). This color change served as a visual indicator of the ongoing crosslinking process [37].

The successful formation of stable crosslinked networks was verified through both visual and mechanical assessments. The standardized grid pattern background revealed consistent transparency across all light intensities while highlighting the varying degrees of RFP photobleaching (Figure 1b). Furthermore, a simple tilting test demonstrated the mechanical integrity of the photocrosslinked hydrogels: while the non-irradiated control group remained in a liquid state and flowed freely, all photocrosslinked samples maintained their hemispherical shape regardless of the applied light intensity (Figure 1c). This observation confirms the successful establishment of crosslinked networks across all tested light intensities, though the rate of network formation varied with light intensity as evidenced by the differences in RFP photobleaching. These results demonstrate that stable collagen hydrogels can be reliably fabricated across a wide range of light intensities (10 K–100 K lux), providing flexibility in the manufacturing process while maintaining structural integrity.

### 3.2. Structural Characteristics of Photocrosslinked Hydrogels at Various Light Intensities

**Figure 2:**
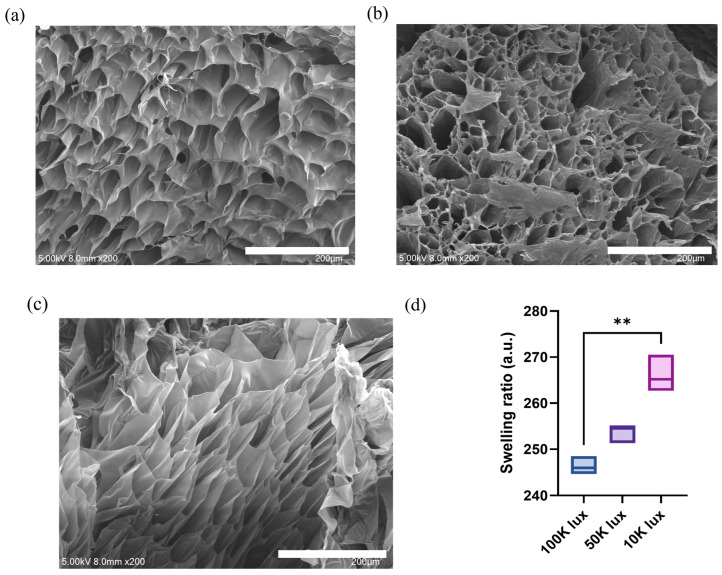
Characterization of photocrosslinked hydrogels prepared at different light intensities. (**a**–**c**) Lyophilized cross-section SEM images of hydrogels prepared at different light intensities: (**a**) 100 K lux, (**b**) 50 K lux, and (**c**) 10 K lux (scale bar: 200 μm). (**d**) Swelling ratio of hydrogels after 24 h of photocrosslinking (** *p* < 0.01) (*n* = 3).

The microstructural characteristics of photocrosslinked hydrogels were examined through SEM imaging of lyophilized cross-sections and swelling ratio measurements (Figure 2). SEM analysis revealed distinct structural variations among hydrogels prepared under different light intensities, demonstrating the significant impact of crosslinking kinetics on network formation. At 100 K lux, the hydrogels exhibited uniform and densely packed porous networks with well-defined pore boundaries, indicating efficient and homogeneous crosslinking (Figure 2a). Hydrogels formed at 50 K lux maintained structural integrity while showing pores ranging from 20 to 100 μm with heterogeneous distribution (Figure 2b). The 10 K lux samples developed more loosely organized networks characterized by larger pores (Figure 2c).

These structural variations directly correlate with the crosslinking kinetics governed by light intensity. Under high light intensity (100 K lux), rapid and uniform photoinitiator activation leads to simultaneous crosslinking throughout the material, resulting in homogeneous network formation. In contrast, lower light intensity (10 K lux) induces gradual crosslinking, allowing more time for structural reorganization during network formation, which contributes to the observed differences in network architecture.

The distinct network architectures significantly influenced the hydrogels’ water absorption capacity, as demonstrated by the 24 h swelling ratio analysis (Figure 2d). A clear inverse relationship emerged between light intensity and swelling ratio, with 10 K lux hydrogels exhibiting a significantly higher swelling ratio (266 ± 4-fold) compared to 100 K lux samples (246 ± 2-fold). This enhanced swelling capacity in low-intensity conditions can be attributed to the looser network architecture and larger pores, which facilitate greater water retention. The observed relationship between light intensity, network structure, and swelling behavior provides a simple yet effective means to tailor hydrogel properties for specific applications, balancing water absorption capacity with structural stability.

### 3.3. Rheological Properties of Photocrosslinked Hydrogels

**Figure 3:**
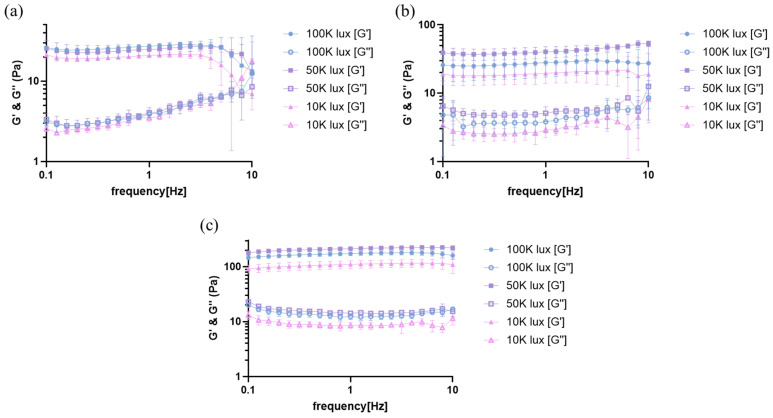
Effect of light intensity on rheological properties of photocrosslinked hydrogels. (**a**) Rheological properties of hydrogels immediately after photocrosslinking. (**b**) Rheological properties of hydrogels after 24 h of photocrosslinking. (**c**) Rheological properties of hydrogels after 24 h of swelling following photocrosslinking. G′ and G″ represent storage and loss moduli, respectively.

The viscoelastic properties of photocrosslinked hydrogels were characterized through frequency sweep measurements at three conditions: immediately after crosslinking, after 24 h of stabilization, and in the swollen state (Figure 3). Immediately after crosslinking (Figure 3a), all samples displayed characteristic hydrogel behavior, with storage modulus (G′) exceeding loss modulus (G″) throughout the measured frequency range. At 1 Hz, the storage modulus showed a positive correlation with light intensity: 100 K lux samples exhibited G′ of ~130 Pa, followed by 50 K lux (~105 Pa) and 10 K lux (~95 Pa) conditions, suggesting that higher light intensities initially lead to increased crosslinking density.

The rheological properties evolved significantly over time and under different conditions. After 24 h of stabilization (Figure 3b), the storage modulus at 1 Hz increased to 28 ± 10 Pa, 40 ± 8 Pa, and 20 ± 6 Pa for 100 K, 50 K, and 10 K lux conditions, respectively, showing enhancement from their initial states, indicating continued crosslinking reactions and matrix stabilization post-photoactivation. Interestingly, when subsequently swollen in PBS for 24 h (Figure 3c), the hydrogels exhibited their highest G′ values, contrary to the expected decrease in mechanical properties typically associated with water absorption. This unexpected enhancement in storage modulus can be attributed to molecular interactions, particularly hydrogen bonding, between the water molecules and the collagen matrix. Similar enhancement of mechanical properties through hydrogen bonding networks has been demonstrated in other hydrogel systems, where the formation of dynamic hydrogen bonds contributed to improved mechanical strength [38].

The influence of light intensity on mechanical properties showed distinct patterns across these conditions. The 10 K lux samples consistently exhibited the lowest G′ values, indicating that the lower crosslinking density achieved at this light intensity results in inherently weaker mechanical properties. The 50 K lux condition, while showing intermediate G′ values initially, demonstrated the highest storage modulus in the swollen state, suggesting optimal interaction with absorbed water molecules. This observation correlates with the previously observed swelling behavior, where intermediate light intensity appeared to create a network structure capable of extensive water interactions. In contrast, while 100 K lux samples showed the highest initial G′ values, their relatively lower swelling capacity likely limited water–matrix interactions, resulting in lower storage modulus compared to 50 K lux samples in the swollen state.

These findings reveal the complex interplay between light intensity, network formation, and hydrogel–water interactions in determining the final mechanical properties. While higher light intensity initially produces stiffer networks through increased crosslinking density, the ultimate mechanical properties appear to be significantly influenced by the hydrogel’s capacity for water interactions. This understanding provides valuable insights for tailoring hydrogel properties for specific applications, suggesting that intermediate light intensities might offer an optimal balance between initial network formation and final mechanical properties in hydrated environments.

### 3.4. Cell Viability and Proliferation in Photocrosslinked Hydrogels Under 2D Culture Conditions

**Figure 4:**
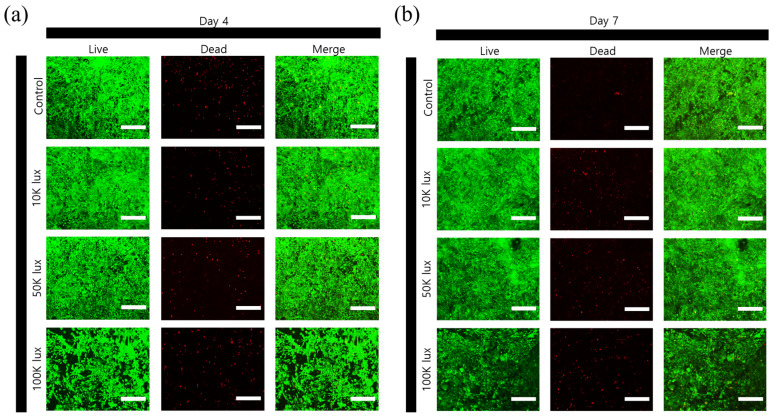

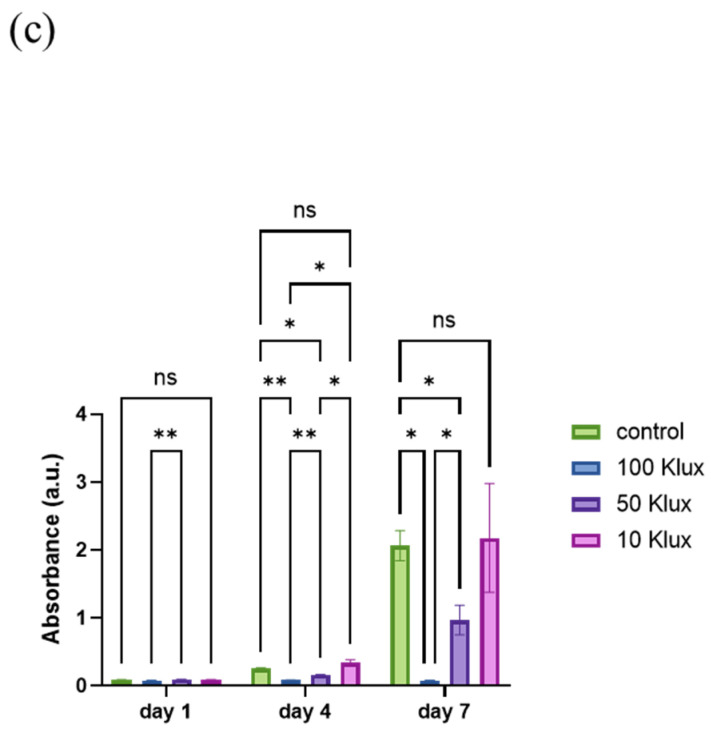
Cell viability and proliferation assessment under different light intensity conditions. Live/dead staining images of 2D cultured cells at (**a**) day 4 and (**b**) day 7, where green fluorescence indicates live cells and red fluorescence indicates dead cells, showing decreased cell density with increasing light intensity (scale bar: 300 μm). (**c**) MTT assay results demonstrating cell proliferation over 7 days, with control and 10 K lux conditions showing significantly higher proliferation by day 7 (ns: not significant, * *p* < 0.05, ** *p* < 0.01) (*n* = 3).

The influence of varying light intensities on cell behavior was evaluated through live/dead staining and MTT assays in 2D culture conditions (Figure 4). Live/dead fluorescence imaging at day 4 (Figure 4a) and day 7 (Figure 4b) demonstrated high cell viability across all conditions, with predominant green fluorescence indicating viable cells and minimal red fluorescence suggesting negligible cell death. Notably, the cell density exhibited an inverse relationship with light intensity, where hydrogels fabricated at lower light intensities supported higher cell populations.

The quantitative assessment of cell proliferation via MTT assay (Figure 4c) revealed distinct patterns of cell growth over time. At day 1, comparable cell viability across all conditions indicated that the photocrosslinking process itself did not induce immediate cytotoxicity. By day 4, the control group and 10 K lux condition showed absorbance values of 0.25 ± 0.02 and 0.34 ± 0.04 a.u., respectively, while 50 K and 100 K lux conditions exhibited lower cell proliferation with absorbance values of 0.16 ± 0.01 and 0.09 ± 0.01 a.u., respectively, although these differences were not statistically significant. This trend became more pronounced by day 7, where both the control and 10 K lux conditions exhibited significantly higher proliferation rates. The 50 K lux condition showed intermediate levels of cell proliferation, significantly lower than the control and 10 K lux groups, while the 100 K lux condition demonstrated the lowest proliferation rate, significantly different from all other groups.

These results reveal critical insights into the relationship between crosslinking intensity and cellular response. While conventional understanding suggests that increased substrate stiffness promotes cell proliferation, our findings showed an opposite trend [39,40,41]. This apparent contradiction can be explained by the complex interplay between mechanical properties and cellular interaction sites. Higher crosslinking densities achieved at 100 K lux created stiffer matrices with more compact network structures, which may physically restrict cell spreading and migration. Additionally, extensive crosslinking can modify cell-adhesive motifs within the collagen structure, particularly affecting the accessibility of integrin-binding sites [42]. This is evidenced by the correlation between mechanical properties and cell morphology, where cells in softer matrices (10 K lux) exhibited more elongated morphologies and enhanced network formation. The intermediate condition (50 K lux) represents a transition point where increased mechanical stability begins to impact cellular behavior, demonstrating that optimal cell response may not necessarily correspond to the highest mechanical strength, but rather to a balance between matrix stability and preservation of cell-interactive domains.

### 3.5. Cell Viability and Proliferation in Photocrosslinked Hydrogels Under 3D Culture Conditions

**Figure 5:**
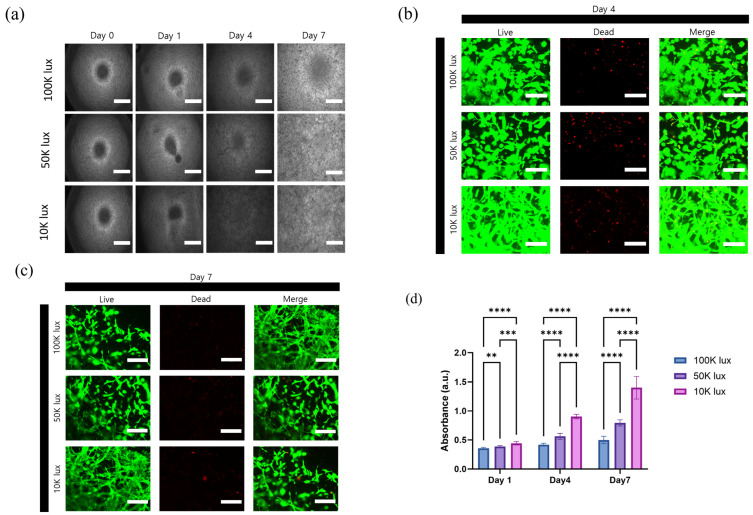
Cell behavior and matrix interactions in 3D photocrosslinked collagen hydrogels. (**a**) Phase contrast microscopy images showing cell morphological changes from day 0 to day 7 (scale bar: 75 μm). Live/dead staining images at (**b**) day 4 and (**c**) day 7, demonstrating cell viability and morphological differences, where green fluorescence indicates live cells and red fluorescence indicates dead cells (scale bar: 300 μm). (**d**) MTT assay results showing significant differences in cell proliferation over 7 days (** *p* < 0.01, *** *p* < 0.001, **** *p* < 0.0001) (*n* = 3).

The cellular response to varying light intensities in 3D photocrosslinked collagen hydrogels was evaluated through multiple analytical approaches (Figure 5). Phase contrast microscopy was employed to track morphological evolution of encapsulated cells from day 0 to day 7 (Figure 5a), revealing distinct patterns of cellular organization across different light intensity conditions. This analysis was complemented by live/dead fluorescence staining at day 4 (Figure 5b) and day 7 (Figure 5c), which demonstrated high cell viability across all conditions as indicated by predominant green fluorescence, with minimal red fluorescence indicating dead cells. Quantitative assessment via MTT assay revealed significant differences in cell proliferation across the tested conditions over 7 days (Figure 5d). The metabolic activity demonstrated a clear inverse relationship with light intensity, with 10 K lux conditions showing the highest proliferation rates and 100 K lux exhibiting the lowest. 

Notably, cells encapsulated in hydrogels prepared under 10 K lux conditions exhibited more elongated morphologies and enhanced cell–cell interactions, forming extensive cellular networks. This morphological transition was particularly evident when comparing across light intensities, with cells in 100 K lux hydrogels maintaining more spherical morphologies, while those in lower light intensity conditions showed progressively increased spreading and network formation. These observations align with the MTT results, suggesting that lower light intensities create microenvironments more conducive to natural cell behavior and tissue-like organization.

These findings highlight the critical relationship between light intensity during photocrosslinking and cellular response in 3D environments. While lower light intensities promote enhanced cellular activity and network formation, practical applications must consider the balance between biological performance and mechanical requirements. This understanding suggests potential strategies for optimization, such as varying light intensities across different regions of a single construct to achieve desired combinations of mechanical and biological properties for specific applications.

Previous studies have demonstrated various approaches to control collagen hydrogel properties, including concentration variation [36], chemical modification [18], and different crosslinking methods [7]. Compared to these methods, our light-intensity-based approach offers unique advantages in terms of experimental simplicity and reproducibility. However, other studies have achieved higher mechanical strengths through multiple crosslinking mechanisms or composite formation, suggesting potential directions for further enhancement of our system [3].

While light intensity modulation provides a straightforward method for controlling hydrogel properties, several limitations should be considered. First, the achievable range of mechanical properties is inherently limited by the single crosslinking mechanism. Second, deep tissue applications may be restricted by light penetration depth, particularly in thick constructs. Third, the inverse relationship between mechanical strength and cell behavior observed in this study suggests that additional parameters may need to be incorporated for applications requiring both high mechanical strength and optimal cell response.

In this study, we focused on demonstrating the precise control of material properties through light intensity modulation and its significant impact on cellular behavior, establishing a fundamental framework for photocrosslinking-based biomaterial design. While our current investigation primarily utilized NIH/3T3 fibroblasts as a model cell type and explored a specific range of light intensities, future studies could further enhance the utility of this approach. For instance, investigating the response of tissue-specific cells or exploring spatially controlled light exposure could expand the application potential of this technique. Additionally, combining this straightforward light-intensity-modulation approach with other strategies, such as the incorporation of bioactive molecules, could provide additional functionality while maintaining the simplicity and effectiveness of the current method. These future directions would build upon the robust foundation established in this study, further advancing the development of precisely controlled biomaterials for tissue engineering applications.

## 4. Conclusions

This study demonstrates a simple yet effective strategy for tailoring collagen hydrogel properties through light intensity modulation during photocrosslinking, revealing distinct correlations between fabrication parameters and material characteristics. Our comprehensive analysis showed that higher light intensities (100 K lux) produced more uniform and compact networks with initially superior mechanical properties, while intermediate intensity (50 K lux) optimized mechanical performance in physiological conditions through balanced crosslinking density and hydrogel–water interactions. Notably, cellular studies revealed that lower light intensities (10 K lux) better preserved collagen’s native cell-interactive domains, leading to enhanced cell proliferation and network formation in both 2D and 3D culture conditions. This approach offers significant advantages over traditional methods by enabling property modification without altering chemical composition, allowing various hydrogels to be created from a single precursor solution.

Looking forward, this light-based fabrication strategy presents several promising research directions. The development of spatially controlled light exposure patterns could enable the creation of gradient properties within a single construct, better mimicking the heterogeneous nature of natural tissues. Additionally, the incorporation of tissue-specific factors while maintaining the simplicity of the current approach could expand its utility in various biomedical applications. These future developments would further advance the potential of light-modulated collagen hydrogels in tissue engineering and drug delivery applications.

## Data Availability

The original contributions presented in this study are included in the article. Further inquiries can be directed to the corresponding authors.
